# Randomised controlled trial to compare the effect of PIOMI (structured) and routine oromotor (unstructured) stimulation in improving readiness for oral feeding in preterm neonates

**DOI:** 10.3389/fped.2023.1296863

**Published:** 2023-11-16

**Authors:** Pari Singh, Nandini Malshe, Aditya Kallimath, Reema Garegrat, Arjun Verma, Nandini Nagar, Rajesh Maheshwari, Pradeep Suryawanshi

**Affiliations:** ^1^ Department of Neonatology, Bharati Vidyapeeth Deemed University, Pune, India; ^2^ Department of Neonatology, Cloudnine Hospital, Bengaluru, India; ^3^ Department of Neonatology, Westmead Hospital, Westmead, NSW, Australia

**Keywords:** neonate, oral motor stimulation, prematurity, oral feeding readiness, exclusive breastfeeding

## Abstract

**Background:**

Oral motor stimulation interventions improve oral feeding readiness and earlier full oral feeding in preterm neonates. However, using a structured method may improve the transition time to full oral feeds and feeding efficiency with respect to weight gain and exclusive breastfeeding when compared to an unstructured intervention.

**Objective:**

To compare the effect of Premature Infant Oral Motor Intervention (PIOMI) and routine oromotor stimulation (OMS) on oral feeding readiness.

**Methods:**

Randomised controlled trial conducted in a neonatal intensive care unit between June-December 2022. Preterm neonates, 29^+0^–33^+6^ weeks corrected gestational age, were studied. The intervention group received PIOMI and the control group received OMS. Primary outcome: time to oral feeding readiness by Premature Oral Feeding Readiness Assessment Scale **(**POFRAS) score ≥30. Secondary outcomes: time to full oral feeds, duration of hospitalisation, weight gain, and exclusive breastfeeding rates.

**Results:**

A total of 84 neonates were included and were randomised 42 each in PIOMI and OMS groups. The mean chronological age and time to oral feeding readiness were lower by 4.6 and 2.7 days, respectively, for PIOMI. The transition time to full oral feeds was 2 days lower for PIOMI and the duration of hospitalisation was 8 days lower. The average weight gain was 4.9 g/kg/day more and the exclusive breastfeeding rates at 1 month and 3 months post-discharge were higher by 24.5% and 27%, respectively, for the PIOMI group. The subgroup analysis of study outcomes based on sex and weight for gestational age showed significant weight gain on oral feeds in neonates receiving PIOMI. Similarly, the subgroup analysis based on gestational age favoured the PIOMI group with significantly earlier transition time and weight gain on oral feeds for the neonates >28 weeks of gestational age. The odds of achieving oral feeding readiness by 30 days [OR 1.558 (0.548–4.426)], full oral feeds by 45 days [OR 1.275 (0.449–3.620)], and exclusive breastfeeding at 1 month [OR 6.364 (1.262–32.079)] and 3 months [3.889 (1.186–12.749)] after discharge were higher with PIOMI.

**Conclusion:**

PIOMI is a more effective oromotor stimulation method for earlier and improved oral feeding in preterm neonates.

**Clinical trial registration:**

https://ctri.nic.in/Clinicaltrials/pdf_generate.php?trialid=70054&EncHid=34792.72281&modid=1&compid=19','70054det', identifier, CTRI/2022/06/043048.

## Introduction

Annually, approximately 15 million neonates are born prematurely (1) with a high risk for oral feeding difficulties due to uncoordinated suck swallow reflexes and poor oral muscle tone (2, 3). Therapies for early attainment of oral feeding are oromotor stimulation (OMS) techniques such as intraoral, perioral stroking and non-nutritive sucking (NNS), Beckman's Oral Motor Intervention (BOMI), and Premature Infant Oral Motor Intervention (PIOMI). PIOMI is a 5-minute 8-step therapy focusing on the lip, jaw, and tongue movements. It simulates the in-utero oral motor experience and has been reported to result in a faster transition to full oral feeds, improved suck strength, and increased breastfeeding rates (4). A study by Arora et al. suggested that PIOMI improves the oromotor skills documented as improved mean Neonatal Oromotor Assessment Scale (NOMAS) scores in preterm neonates (5). As a part of feeding rehabilitation, Ghomi et al. reported the earlier introduction of first oral feeds and shorter hospitalisation with PIOMI (6). A few studies have reported the beneficial effect of PIOMI on feeding efficiency and breastfeeding rates but a statistically significant inference could not be drawn from these (7–9). Hence, conflicting evidence exists for the said outcomes and there is a paucity of data for comparison between structured and unstructured methods of oral motor stimulation. This study was, therefore, designed to test the effectiveness of PIOMI over routine OMS on oral feeding readiness.

## Methodology

A single-centre randomised controlled trial was conducted in a tertiary-level neonatal intensive care unit enrolling neonates between June to December 2022. All preterm neonates with birth gestation of <34 weeks and corrected gestational age (CGA) 29^+0^–33^+6^ weeks who were free of invasive ventilation and inotropic support were assessed for eligibility. Neonates with a neuromuscular disorder, chromosomal anomaly, or craniofacial malformation were excluded. Any neonate with maternal retroviral disease was excluded from the outcome analysis for exclusive breastfeeding. Written informed consent was taken from parents prior to enrolment. Maternal and neonatal baseline characteristics (mode of delivery, indication of delivery, gestational age, sex, birth weight, and resuscitation details) were recorded. Included neonates were randomised into two groups, namely, PIOMI and routine OMS in a 1:1 ratio by simple randomisation using a computer-generated random number table, and intervention was started after 29^+0^ weeks CGA.

The baseline Premature Oral Feeding Readiness Assessment Scale (POFRAS) assessment was done before the initiation of intervention by one of the two independent blinded scorers (Supplementary file). The blinding of the study participants and intervention providers could not be done, however, the intervention providers and POFRAS scorers were blinded to each other. Neonates who were randomised to the PIOMI group were administered intervention 15 min before the gavage feed once daily using all aseptic precautions and with gloved fingers if CGA was 29^+0^–30^+6^ weeks. This process was continued for 7 days until the next POFRAS assessment. After CGA 31 weeks, PIOMI was similarly performed twice daily. PIOMI was done by the principal investigator who underwent training under the founder of PIOMI prior to the commencement of the study. The other group received OMS from trained nursing staff.

For PIOMI, the neonate was positioned in the midline position with the neck slightly flexed and the chin tucked in. Following this, the neonate underwent one cycle each of cheek C-stretch, lip roll, lip curl/stretch, and gum massage for 30 s each. This was followed by stretching of lateral borders of the tongue/cheek for 15 s and mid-blade of the tongue/palate for 30 s. After this, elicitation of suck was performed for 15 s followed by non-nutritive sucking on the mother's breast (or gloved finger/pacifier if the mother was not available) for 2 min. The entire process lasted 5 min.

The second group received OMS as part of routine care. This was a 15-min, 3-step technique comprising of two finger circular movements in a U-shaped fashion from both ears, followed by O-shaped perioral stimulation and ending with pouting stimulation of the cheeks. This method was done 15 min prior to each gavage feed by a trained nurse.

In both groups, the intervention was suspended if there was sudden heart rate acceleration/deceleration, desaturation, apnoea, hiccupping, yawning, sneezing, frowning, looking away, squirming, frantic/disorganised activity, pushing away of arms and legs or if the neonate became sick in the intervening period and required invasive ventilator support/inotropes. The intervention was resumed after 24 h of resolution of the issue and continued for the subsequent 7 days.

Each POFRAS assessment was done after 7 days of intervention. If the score was <25 in either group, the respective intervention was repeated for another 7 days and POFRAS was reassessed. If the score was 25–29 in either group, the respective intervention was repeated for another 3 days and POFRAS was reassessed. Oral feeds were started after POFRAS ≥30. Upon tolerance, feeds were increased gradually to full oral feeds. Exclusive breastfeeding was assessed at 1 month and 3 months post-discharge in both groups. Exclusive breastfeeding was defined as feeding the infant only breastmilk from his or her mother until the time of assessment and no other solids or liquids except for drops or syrups containing vitamins, minerals, supplements, or medicines.

The primary outcome measure was time to oral feed readiness and secondary outcome measures were transition time to full oral feeds, duration of hospital stay, weight gain, and exclusive breastfeeding rate post-discharge.

The study conformed to the reporting checklist criteria for randomised trial based on the CONSORT guidelines.

## Statistical analysis

Data was entered in a Microsoft Excel spreadsheet (Microsoft Corp, Redmond, WA, USA) and analysed using IBM SPSS statistical software version 25. Continuous variables were expressed as mean (standard deviation) or median (inter-quartile range), depending on the distribution of the data. Categorical variables were expressed using frequencies and percentages. For qualitative data variables, the Chi-square test was used and for quantitative data variables, two independent sample t and median tests were used. *P*-value < 0.05 was considered significant. Kaplan–Meier probability analysis curves were used for the establishment of oral feeds. An odds ratio analysis was performed for outcomes related to oral feeding and exclusive breastfeeding. Intention to treat and per protocol analysis was done for exclusive breastfeeding rates. For the outcomes related to the progression of feeds and weight, a subgroup analysis was conducted for sex, gestational age, and weight for gestational age. The inter-observer variability was calculated to be 0.933 (Cronbach's alpha) between the two independent blinded scorers on 20 subjects prior to the commencement of the study. The sample size calculated for statistical significance as per the feeding outcome of a previous study (5) was 42 with 21 in each group.

## Results

**Figure 1:**
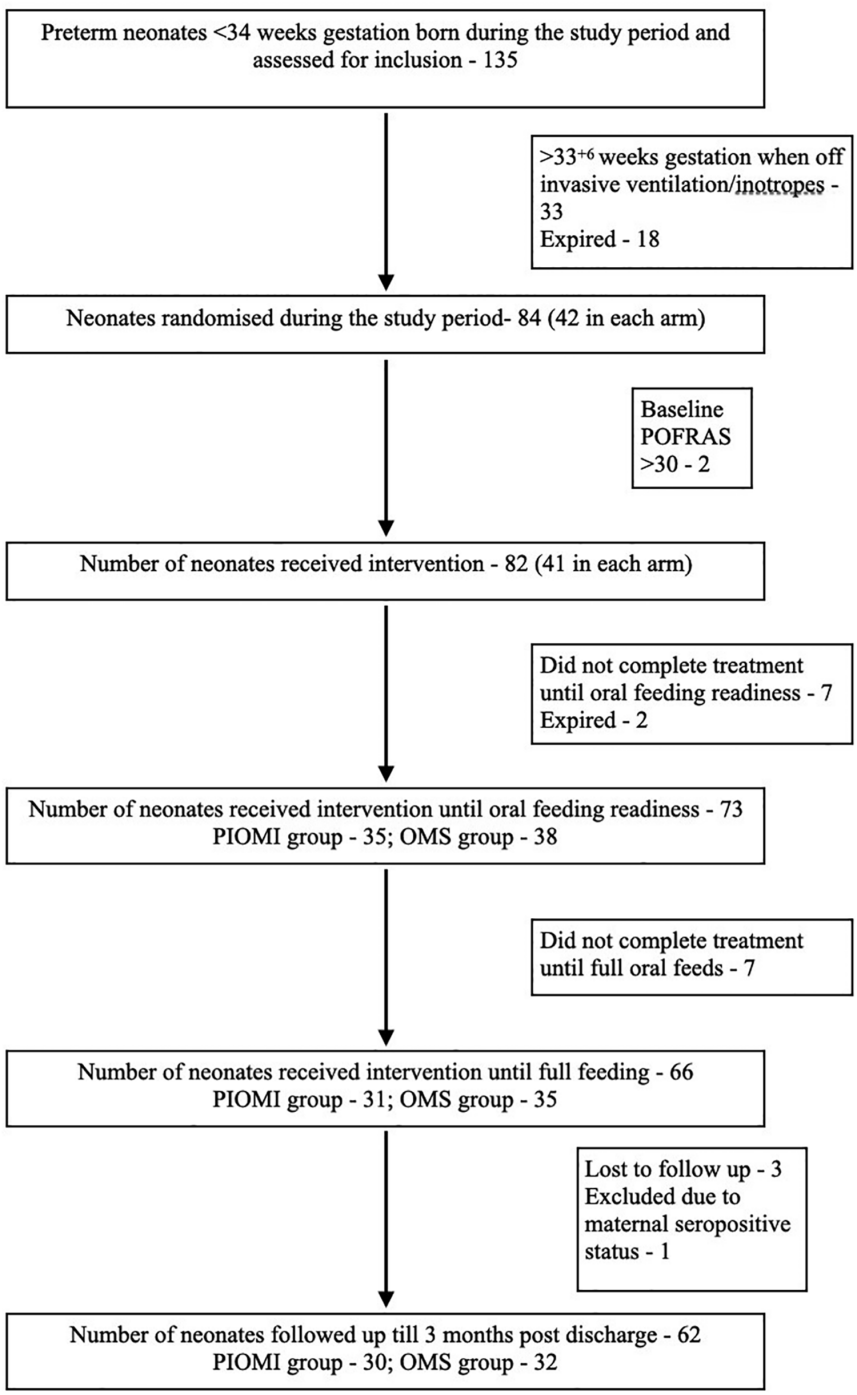
Flowchart of the study.

The study included 84 neonates divided into two groups of 42 each to receive either PIOMI or routine OMS. The study flowchart is described in Figure 1. At birth, the mean gestational age (GA) of the neonates in the PIOMI and OMS groups was 30.6 and 30.3 weeks, respectively, and the mean birthweight was 1,304 and 1,372 g, respectively. The maternal and neonatal characteristics were comparable for both groups (Table 1).

**Table 1:** Birth characteristics of the study sample.

	PIOMI [*n* = 42] (%)	Routine OMS [*n* = 42] (%)	*P*-value
Mode of delivery			
LSCS	32 (76.2)	30 (71.4)	0.621
Vaginal	10 (23.8)	12 (28.6)	
Indication of delivery			
Premature labor/rupture of membranes	21 (50)	26 (61.9)	0.505
Severe pre-eclampsia/eclampsia	10 (23.8)	6 (14.3)	
Antenatal ultrasound Doppler changes	5 (11.9)	5 (11.9)	
Placenta previa/Abruptio placentae	5 (11.9)	4 (9.5)	
Severe oligohydramnios	1 (2.4)	1 (2.4)	
Parity			
Primiparous	28 (67)	32 (76.2)	0.352
Multiparous	14 (33)	10 (23.8)	
Gestational age			
<28 weeks	3 (7.1)	4 (9.5)	0.570
28–31 + 6 weeks	26 (61.9)	27 (64.3)	
32–33 + 6 weeks	13 (31)	11 (26.2)	
Mean gestational age in weeks	30.6 ± 1.66	30.3 ± 1.85	
Sex			
Male	21 (50)	25 (59.5)	0.383
Female	21 (50)	17 (40.5)	
Birth weight			
<1,000 g	9 (21.4)	7 (16.6)	0.525
1,000–1,499 g	22 (52.4)	22 (52.4)	
>1,500 g	11 (26.2)	13 (31)	
Mean weight in grams	1,304 ± 350	1,372 ± 333	0.365
Weight for gestational age			
SGA	8 (19)	5 (11.9)	0.368
AGA	34 (81)	37 (88.1)	
Mean 5-min APGAR	8.2 ± 1.2	7.97 ± 1.1	0.363

PIOMI, premature infant oral motor intervention; OMS, oro-motor stimulation; LSCS, lower segment caesarean section; SGA, small for gestational age; AGA, appropriate for gestational age.

The mean CGA in both groups at the start of intervention was 31.4 weeks (*P*- 0.851) and the mean birthweight was 1,245 and 1,323 g (*P*- 0.256), respectively, for PIOMI and OMS.

Although the CGA at POFRAS score ≥30 was similar for both groups, the chronological age was lower by 4.6 days for the PIOMI group (*P*- > 0.05) and these neonates achieved oral feeding readiness 2.7 days earlier (*P*- > 0.05) (Table 2). This primary outcome was assessed for 73 neonates who completed treatment until oral feeding readiness was achieved.

**Table 2:** Characteristics of the study sample at the time of feeding readiness and full oral feeds.

	PIOMI (*n* = 35)	Routine OMS (*n* = 38)	*P*-value
Mean gestational age in weeks at score ≥30	34.1 ± 1	34.3 ± 1.5	0.542
Mean age at score ≥30, days of life	24.7 ± 13.6	29.3 ± 18.3	0.233
Median [IQR] age at score ≥30, days of life	21 [14, 31]	22 [15, 40]	0.911
Mean number of days from the start of the intervention to score ≥30	18.6 ± 11.6	21.3 ± 13.6	0.374
Median [IQR] number of days for score ≥30	16 [10, 28]	16 [11, 34]	0.911
	PIOMI (*n* = 31)	Routine OMS (*n* = 35)	*P*-value
Mean gestational age in weeks at full oral feeds	35.9 ± 1.22	36.4 ± 1.9	0.248
Mean age at full oral feeds, days of life	38.3 ± 15.1	44.4 ± 23.4	0.210
Median [IQR] age at full oral feeds, days of life	37 [26, 48]	35 [27, 69]	0.468
Mean number of days from the start of oral feeds to full oral feeding	9.1 ± 2.2	11.1 ± 3.3	0.007
Mean weight in grams at full oral feeds	1,862 ± 185	1,874 ± 201	0.8
Average duration of hospital stay in days	37.06 ± 16.2	45.1 ± 23.1	0.104
Median [IQR] duration of hospital stay in days	38 [27, 48]	36 [27, 69]	1
Average weight gain [g/kg/day]	14.6 ± 3.7	9.7 ± 2.9	0.0001

PIOMI, premature infant oral motor intervention; OMS, oro-motor stimulation; PMA, post menstrual age; IQR, inter-quartile range.

**Figure 2:**
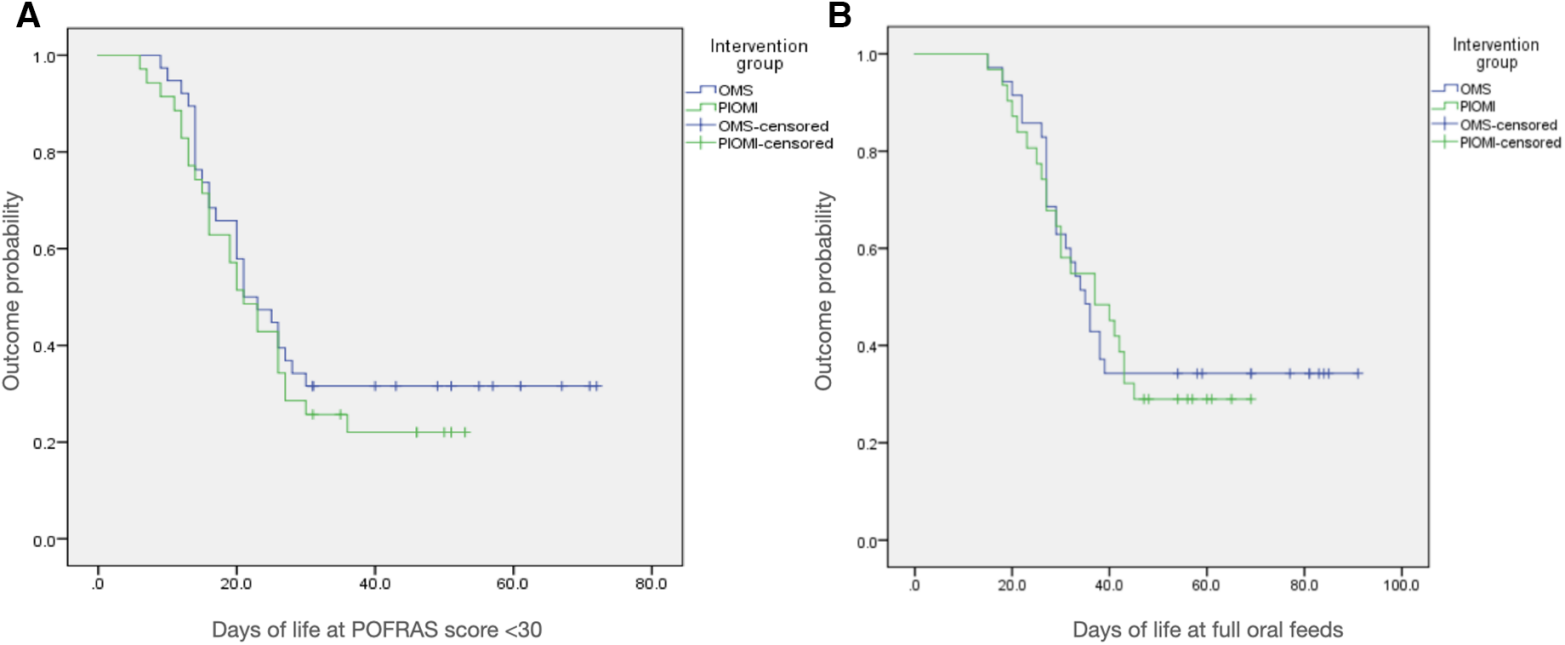
Kaplan-Meier analysis curves for the probability of (**A**) not achieving oral feeding readiness by 30 days of life and (**B**) not achieving full oral feeds by 45 days of life.

As per Kaplan–Meier analysis, the probability of not achieving oral feeding readiness by 30 days of life (DOL) was lower for the PIOMI group (Figure 2A).

Out of 66 neonates followed until discharge, the CGA at full oral feeds was 35.9 and 36.4 weeks, respectively, for PIOMI and OMS (*P*- > 0.05). The age for full oral feeds was 6.1 DOL lower, the transition time from initiation to full oral feeds was 2 days less, and the duration of hospitalisation was 8 days less for the PIOMI group (*P*- > 0.05). The average weight gain was higher by 4.9 g/kg/day with PIOMI (*P*- < 0.05) (Table 2).

As per Kaplan–Meier analysis, the probability of not achieving full oral feeds by 45 DOL was lower for the PIOMI group (Figure 2B).

A subgroup analysis for the progression of feeding characteristics of the study sample from birth till the achievement of full oral feeds was done for sex and weight for gestational age (Table 3). A statistically significant result could only be achieved for average weight gain from initiation to achievement of full oral feeds.

**Table 3:** Progression of feeding characteristics of the study sample from birth till achievement of full feeds based on sex and weight for gestational age.

	PIOMI (*n* = 42)		Routine OMS (*n* = 42)		*P*-value
Mean gestational age in weeks at birth	30.6 ± 1.66		30.3 ± 1.85		0.436
Male AGA	*n* = 19	30.8 ± 1.4	*n* = 21	30.4 ± 2.1	0.487
Male SGA	*n* = 2	29.3	*n* = 4	30.9 ± 1.8	0.333
Female AGA	*n* = 15	30.5 ± 2.2	*n* = 16	30.2 ± 1.6	0.666
Female SGA	*n* = 6	30.8 ± 1	*n* = 1	28.6	–
Mean weight in grams at birth	1,304 ± 350		1,372 ± 333		0.365
Male AGA	*n* = 19	1,401 ± 295	*n* = 21	1,424 ± 345	0.823
Male SGA	*n* = 2	800 ± 141	*n* = 4	1,140 ± 209	0.113
Female AGA	*n* = 15	1,371 ± 377	*n* = 16	1,401 ± 301	0.784
Female SGA	*n* = 6	996 ± 165	*n* = 1	745	–
	PIOMI (*n* = 35)		Routine OMS (*n* = 38)		*P*-value
Mean gestational age in weeks at score ≥30	34.1 ± 1		34.3 ± 1.5		0.542
Male AGA	*n* = 17	33.8 ± 1.05	*n* = 19	34.3 ± 1.1	0.173
Male SGA	*n* = 1	34.5	*n* = 4	35.8 ± 1.8	–
Female AGA	*n* = 12	34.1 ± 0.8	*n* = 14	33.5 ± 0.9	0.087
Female SGA	*n* = 5	34.9 ± 1	*n* = 1	39	–
Mean number of days from the start of the intervention to score ≥30	18.6 ± 11.6		21.3 ± 13.6		0.374
Male AGA	*n* = 17	17.2 ± 11.1	*n* = 19	21.4 ± 13.9	0.327
Male SGA	*n* = 1	33	*n* = 4	21.5 ± 10.4	–
Female AGA	*n* = 12	19 ± 13.6	*n* = 14	18.4 ± 10.9	0.901
Female SGA	*n* = 5	19.2 ± 11.6	*n* = 1	58	–
	PIOMI (*n* = 31)		Routine OMS (*n* = 35)		*P*-value
Mean gestational age in weeks at full oral feeds	35.9 ± 1.22		36.4 ± 1.9		0.248
Male AGA	*n* = 15	35.8 ± 1.3	*n* = 18	36.3 ± 1.9	0.394
Male SGA	*n* = 1	36.2	*n* = 4	37.6 ± 1.9	–
Female AGA	*n* = 11	35.6 ± 0.8	*n* = 12	35.9 ± 1.5	0.561
Female SGA	*n* = 4	37.2 ± 1.3	*n* = 1	40.5	–
Mean number of days from the start of oral feeds to full oral feeding	9.1 ± 2.2		11.1 ± 3.3		0.007
Male AGA	*n* = 15	8.7 ± 2.5	*n* = 18	9.9 ± 2.4	0.170
Male SGA	*n* = 1	10	*n* = 4	11.8 ± 1.5	–
Female AGA	*n* = 11	9.8 ± 2	*n* = 12	12.5 ± 4.4	0.076
Female SGA	*n* = 4	8.8 ± 1.3	*n* = 1	13	–
Mean weight in grams at full oral feeds	1,862 ± 185		1,874 ± 201		0.8
Male AGA	*n* = 15	1,888 ± 187	*n* = 18	1,931 ± 220	0.554
Male SGA	*n* = 1	1,875	*n* = 4	1,731 ± 108	–
Female AGA	*n* = 11	1,902 ± 173	*n* = 12	1,845 ± 177	0.444
Female SGA	*n* = 4	1,650 ± 103	*n* = 1	1,750	–
Average weight gain [g/kg/day]	14.6 ± 3.7		9.7 ± 2.9		0.0001
Male AGA	*n* = 15	13.6 ± 3.7	*n* = 18	8.6 ± 2.6	0.0001
Male SGA	*n* = 1	13.1	*n* = 4	10.5 ± 4.1	–
Female AGA	*n* = 11	15.8 ± 3.7	*n* = 12	11.2 ± 2.4	0.0018
Female SGA	*n* = 4	15.5 ± 3.5	*n* = 1	7.7	–

PIOMI, premature infant oral motor intervention; OMS, oro-motor stimulation; AGA, appropriate for gestational age; SGA, small for gestational age.

The subgroup analysis for the feeding characteristics based on gestational age (Table 4) favoured the PIOMI group, however, a statistically significant inference could only be drawn for transition to full oral feeds for neonates 28^+0^–31^+6^ weeks gestation and average weight gain on oral feeds for neonates >28 weeks gestational age.

**Table 4:** Progression of feeding characteristics of the study sample from birth till achievement of full feeds based on gestational age.

	PIOMI (*n* = 35)		Routine OMS (*n* = 38)		*P*-value
Mean gestational age in weeks at score ≥30	34.1 ± 1		34.3 ± 1.5		0.542
<28 weeks	*n* = 2	34.6 ± 0.8	*n* = 4	35.2 ± 0.5	0.305
28–31 + 6 weeks	*n* = 24	34.1 ± 1.1	*n* = 25	34.2 ± 1.8	0.816
32–33 + 6 weeks	*n* = 9	33.9 ± 0.6	*n* = 9	34.2 ± 0.2	0.174
Mean number of days from the start of the intervention to score ≥30	18.6 ± 11.6		21.3 ± 13.6		0.374
<28 weeks	*n* = 2	39.5 ± 0.7	*n* = 4	41.8 ± 4.6	0.543
28–31 + 6 weeks	*n* = 24	20.9 ± 10.3	*n* = 25	22 ± 12.6	0.740
32–33 + 6 weeks	*n* = 9	7.7 ± 3.6	*n* = 9	9.8 ± 3.1	0.203
	PIOMI (*n* = 31)		Routine OMS (*n* = 35)		*P*-value
Mean gestational age in weeks at full oral feeds	35.9 ± 1.22		36.4 ± 1.9		0.248
<28 weeks	*n* = 2	36.1 ± 1.2	*n* = 4	38.4 ± 1.1	0.077
28–31 + 6 weeks	*n* = 22	36.1 ± 1.3	*n* = 22	36.4 ± 2.1	0.572
32–33 + 6 weeks	*n* = 7	35.4 ± 0.7	*n* = 9	35.7 ± 0.7	0.417
Mean number of days from the start of oral feeds to full oral feeding	9.1 ± 2.2		11.1 ± 3.3		0.007
<28 weeks	*n* = 2	11 ± 1.4	*n* = 4	13.5 ± 3.1	0.357
28–31 + 6 weeks	*n* = 22	8.9 ± 2	*n* = 22	10.7 ± 2.7	0.016
32–33 + 6 weeks	*n* = 7	9.2 ± 2.8	*n* = 9	11.1 ± 4.4	0.338
Mean weight in grams at full oral feeds	1,862 ± 185		1,874 ± 201		0.8
<28 weeks	*n* = 2	1,925 ± 134	*n* = 4	2,107 ± 358	0.544
28–31 + 6 weeks	*n* = 22	1,845 ± 199	*n* = 22	1,842 ± 174	0.958
32–33 + 6 weeks	*n* = 7	1,897 ± 164	*n* = 9	1,847 ± 114	0.483
Average weight gain [g/kg/day]	14.6 ± 3.7		9.7 ± 2.9		0.0001
<28 weeks	*n* = 2	12.3 ± 1.1	*n* = 4	9.8 ± 2.2	0.219
28–31 + 6 weeks	*n* = 22	14.5 ± 3.7	*n* = 22	9.9 ± 3.1	0.0001
32–33 + 6 weeks	*n* = 7	15.6 ± 4.2	*n* = 9	9 ± 2.8	0.002

PIOMI, premature infant oral motor intervention; OMS, oro-motor stimulation.

A total of 62 neonates were assessed for breastfeeding till 3 months post-discharge. The exclusive breastfeeding rate was higher by 24.5% (*P*- 0.015) at 1-month post-discharge in the PIOMI group (per protocol analysis) and by 14.37% (*P*- 0.185) (per intention to treat analysis). At 3 months post-discharge, 27% (*P*- 0.022) more neonates in the PIOMI group were on exclusive breastfeeding (per protocol analysis) and 16.6% (*P*- 0.128) (per intention to treat analysis).

**Figure 3:**
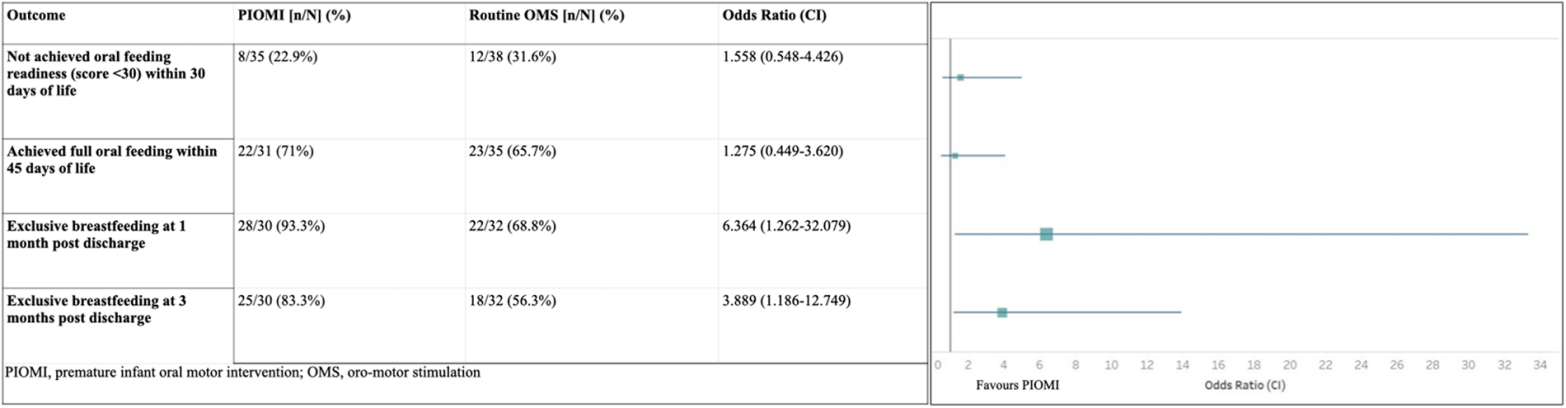
Outcome analysis based on the odds ratio.

Although not statistically significant, the odds of achieving oral feeding readiness and establishment of full oral feeds were higher in the PIOMI group. The odds of exclusive breastfeeding at 1 and 3 months post-discharge were significantly higher in the PIOMI group as compared to OMS (Figure 3).

## Discussion

In this study comparing the effect of two methods of oromotor stimulation on readiness for oral feeding in preterm neonates, we did not find a significant difference. We, however, noted the significantly earlier transition from initiation to full oral feeds, better weight gain, and post-discharge breastfeeding rates in the structured method of oromotor stimulation (PIOMI).

Our study had comparable baseline characteristics with the previous studies (5, 8, 10). Most of the neonates in both groups were of GA 28^+0^–31^+6^ weeks with a birthweight ranging from 1,000 to 1,499 g, which was appropriate for GA. However, as opposed to the previous studies (5, 8, 10), in which, intervention was started after attaining a pre-specified gavage feeding volume, in our study, the neonates were included as soon as they were clinically and hemodynamically stable and a minimum feeding volume was not considered necessary for beginning the intervention. Furthermore, most of the previous studies compared PIOMI to routine care where the control groups had not received any form of oral motor stimulation.

Both the intervention groups achieved oral feeding readiness at a similar CGA and a trend for lower chronological age for oral feeding readiness was noted with PIOMI but this was not statistically significant. The probability of achieving oral feeding readiness by 30 DOL was higher with PIOMI.

Similar to our observation, Sumarni et al. (11) evaluated oral feeding readiness by POFRAS score before and after administration of 7 days of oral motor stimulation and observed that the neonates receiving PIOMI had a higher increment in post-POFRAS scores but the result was not statistically significant. Variability in statistical significance has also been observed for this outcome in other studies, most of which did not utilise any feeding readiness assessment tool (8, 10, 12).

The lower CGA and chronological age for achieving full oral feeds for the PIOMI group was not statistically significant, however, a significantly faster transition to full oral feeding was observed with PIOMI. The probability of achieving full oral feeds by 45 DOL was also higher with PIOMI. Some of the previous studies have also suggested a shorter transition time to full oral feeds using PIOMI (5, 6, 10, 13, 14). The difference in the days to independent oral feeding as compared to the present study could be attributed to variable methodology.

Neonates in the PIOMI group in our study could be discharged 8 days earlier. Similarly, Arora et al. (5) reported that neonates receiving PIOMI could be discharged earlier as compared to sham intervention (P > 0.05). Some of the other studies have observed a statistically significant reduction in the duration of stay with PIOMI, however, the control groups in these studies had not received any form of oral motor stimulation (4, 6, 8, 12, 14–16).

Although the difference between the groups for oral feeding readiness, full oral feeding, and duration of hospitalisation was not statistically significant, each day saved in terms of clinical management has a significant implication on the expenditure for the affected family and the healthcare system. Earlier initiation and achievement of oral feeds will help to establish the emotional bond between the mother-infant dyad and enhance the mother's confidence in feeding and taking care of the neonate. Decreased duration of hospitalisation would reduce the financial burden on the family, especially in a low-middle income setting. Additionally, this will have a profound effect on the available health resources and the economics of the health structure.

The neonates receiving PIOMI had significantly higher weight gain on oral feeds in our study. A similar observation was noted in the study by Thakkar et al. (10). This may indirectly be indicative of better oral feeding efficiency and milk volume transferred in each feed, as has been suggested by some previous studies (7, 10, 17) in favour of PIOMI. However, overall the results have been variable (6, 8). This could be attributed to not following a feeding readiness assessment scale across the previous studies, which may have subjectively altered the judgement of feeding efficiency.

While the subgroup analysis as per sex, weight for gestational age, and gestational age did not reveal statistically significant differences for all study outcomes, it favoured the PIOMI group, particularly the neonates >28 weeks gestation. The results of individual analyses, however, need interpretation, taking into account the small sample size.

The exclusive breastfeeding rates in the present study were significantly higher after discharge in the neonates who had received PIOMI per protocol (p < 0.05). The OR analysis of the same also favored PIOMI, with statistical significance. This finding supports that PIOMI improves the feeding efficiency in preterm neonates (7, 10, 17). Sasmal et al. (8) observed higher breastfeeding rates with PIOMI at 1 month after discharge. Skaaning et al. (9) evaluated the effect of a parent-administered PIOMI-based oral motor stimulation method. Both studies, however, could not establish a significant impact on exclusive breastfeeding with PIOMI. This could be speculated to lower sample size (8), variable methodology, and caregiver-dependent method of PIOMI.

The abovementioned observations suggest that although a structured form of oral motor stimulation may not have resulted in statistically significant differences in initiation and attainment of oral feeds, its effects on improving oral feeding efficiency and exclusive breastfeeding rates are evident.

Our study has several strengths. More than the required number of participants were recruited to achieve statistical power. Both structured and unstructured methods of oral motor stimulation were compared in the present study, thereby, assessing the overall effect of various methods of oral motor stimulation utilised in clinical settings. Additionally, the intervention was started as soon as the neonate was hemodynamically stable, as has been suggested by Lessen et al. (4). The oral feeding was established as per validated scoring systems and not based on subjective assessment. However, the study is not devoid of limitations. It is a single-centre study and, therefore, the findings may not be generalizable. Although the intervention providers were blinded to the weekly POFRAS assessment and scores, the blinding of study participants at the time of providing intervention could not be achieved. The allocation concealment for randomisation was also not performed. Data on maternal education and socio-economic status was not collected, which may have been significant attributing factors for exclusive breastfeeding.

## Conclusion

The neonates receiving PIOMI showed higher weight gain and exclusive breastfeeding rate post-discharge. This suggests improvement in feeding efficiency with a structured intervention. Although a statistically significant difference could not be derived for all outcomes, it may still offer clinical benefit for the patient and the treating facility. We, thus, recommend PIOMI to be more effective for improved oral feeding in preterm neonates 29^+0^–33^+6^ weeks GA. However, multicentric trials with larger sample sizes would be necessary to further strengthen the recommendation.

## Data Availability

The raw data supporting the conclusions of this article will be made available by the authors, without undue reservation.
