# Supplementary figures and images for: Randomised controlled trial to compare the effect of PIOMI (structured) and routine oromotor (unstructured) stimulation in improving readiness for oral feeding in preterm neonates
Source: Front Pediatr. 2023 Nov 16;11:1296863. doi: 10.3389/fped.2023.1296863 (PMC10687572; doi:10.3389/fped.2023.1296863)

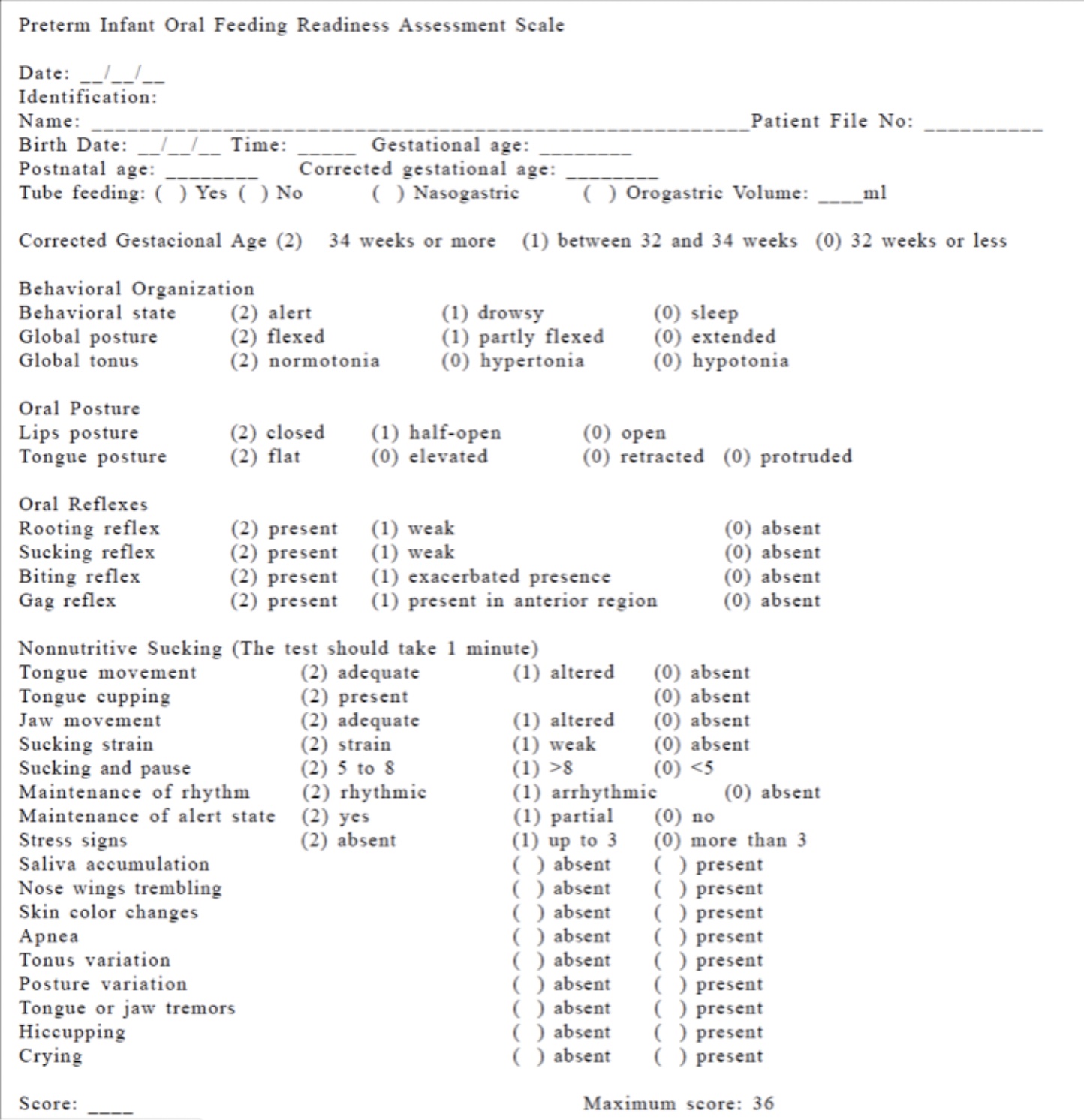

Supplement: Supplementary file 2 [file Image1.jpeg]
